# Complete Genome Sequence of *Bordetella pertussis* Pelita III, the Production Strain for an Indonesian Whole-Cell Pertussis Vaccine

**DOI:** 10.1128/genomeA.00235-17

**Published:** 2017-04-27

**Authors:** Yusuf Sofyan Efendi, Dwi Susanti, Erman Tritama, Michelle Lueders Pasier, Gilang Nadia Niwan Putri, Sugeng Raharso, Iskandar, Pingkan Aditiawati, Ernawati Arifin Giri-Rachman, Biswarup Mukhopadhyay, Endang Purwantini

**Affiliations:** a PT Bio Farma (Persero), Bandung, Indonesia; b Department of Biochemistry, Virginia Tech, Blacksburg, Virginia, USA; c Biocomplexity Institute, Virginia Tech, Blacksburg, Virginia, USA; d School of Life Sciences and Technology, Institut Teknologi Bandung, Bandung, Indonesia

## Abstract

PT Bio Farma, the sole World Health Organization-approved Indonesian vaccine producer, manufactures a whole-cell whooping cough vaccine (wP) that, as part of a pentavalent diphtheria-tetanus-pertussis/hepatitis B/*Haemophilus influenzae* b (DTP/HB/Hib) vaccine, is used in Indonesia and many other countries. We report here the whole-genome sequence for *Bordetella pertussis* Pelita III, PT Bio Farma’s wP production strain.

## GENOME ANNOUNCEMENT

PT Bio Farma (Persero) manufactures whole cell pertussis (wP) vaccine using *Bordetella pertussis* strain Pelita III. The antigenic characteristics of *B. pertussis* strains change over time (1–3), and consequently, the monitoring of these features of working seeds is required to generate effective vaccines. Incidentally, the recent revolution in genomics has made whole-genome shotgun sequencing a rapid, accurate, and cost-effective avenue to examine not only the vaccine antigen genes but also additional genes that are key to the production process. However, it depends on the availability of a whole-genome sequence. For these reasons and for a detailed comparison to other pertussis vaccine production strains, the whole-genome sequence of the working seed of *B. pertussis* strain Pelita III was determined.

The sequencing was performed at the University of Delaware Sequencing & Genotyping Center (Newark, DE) on the PacBio RS II platform, employing single-molecule real-time (SMRT) technology (Pacific Biosciences, Menlo Park, CA) (4), yielding 141,140 reads totaling 888,059,822 bases. The *de novo* genome assembly was performed with the Hierarchial Genome Assembly Process (HGAP) workflow of the SMRT Analysis system (4). The circularity of the assembled sequence was tested using Gepard, and the circular sequence was generated with Amos and Minimus2 (5, 6). The final assembly generated a single contig of a 4.1-Mb genome with 141.91× coverage. The initial identification and annotation of genes were performed using the Integrated Microbial Genomes-Expert Review (IMG/ER) platform of the U.S. Department of Energy’s Joint Genome Institute (Walnut Creek, CA, USA) (7). The GenBank annotation utilized the NCBI Prokaryotic Genome Annotation Pipeline (8).

At the genome level, Pelita III was closely related to *Bordetella pertussis* Tohama I (9, 10), a reference strain (11) and major source of pertussis vaccines (3, 12). The nucleotide sequences for each of the pathogenesis genes, including those for the vaccine antigens namely, pertussis toxin (PT), pertactin (PRN), filamentous hemagglutinin (FHA), and fimbriae (FIM), were the same in the two strains (13). The observed differences between two genomes were of two types: (i) additional elements in Pelita III, likely due to transpositions, a tandem duplication of transposase InsO at two locations (bp 44713 to 44663 and bp 698196 to 699146), a DNA element carrying tRNA_Gly_CCC, an ABC transporter substrate-binding protein, and a partial transposase InsO gene (bp 656897 to 6588869); (ii) a two-nucleotide deletion in Tohama I causing a frameshift in a gene for a methyl-accepting chemotaxis sensory transducer with Pas/Pac sensor that is intact in Pelita (bp 1474828 to 1476270).

### Accession number(s).

The genome sequence has been deposited to GenBank under the accession number CP019957.
